# Analysing plate fixation of a comminuted fracture of the proximal ulna in relation to the elbow joint: a finite element study

**DOI:** 10.1186/s13018-025-06031-4

**Published:** 2025-07-28

**Authors:** J. Šafran, T. Pavlacký, P. Marcián, R. Herůfek, R. Veselý

**Affiliations:** 1 Brno University of Technology, Brno, Czech Republic; 2 Masaryk University, Faculty of Medicine, Brno, Czech Republic

**Keywords:** Proximal ulna comminuted fracture, Elbow joint, Olecranon fracture, Finite element analysis, Locking compression plate, Interfragmentary motion

## Abstract

This study investigated the biomechanical behavior of four different screw configurations used to fix comminuted proximal ulna fractures with a locking compression plate (LCP), via a detailed finite element model based on realistic anatomical geometry. The model incorporated realistic anatomical geometry including both cortical and cancellous bone, soft tissue constraints, and loading conditions representing the physiological self-weight of the forearm, with the humerus fixed at its proximal end. The stress distribution on the plate, strain intensity within the bone tissue, and interfragmentary motion (IFM) between fracture fragments were evaluated for each configuration. The results indicate that all the tested configurations provide adequate stability under normal loading conditions, with no risk of material failure. However, excessive stress concentrations were observed in specific screw regions depending on the configuration, particularly when proximal screws anchoring the olecranon (e.g. screws 2 and 3 in Variant 3) were omitted. Strain analysis revealed moderate physiological bone loading across variants, whereas IFM assessment highlighted the importance of securing the coronoid and apical fragments to prevent compromised healing. These findings suggest that a specific reductions in osteosynthetic material, such as omitting certain diaphyseal screws while maintaining crucial olecranon and coronoid fixation, may provide sufficient fracture stabilisation under the modelled conditions, potentially minimising implant-related complications. This modelling approach offers a valuable tool for preclinical assessment of osteosynthesis strategies and supports future comparative research on fixation methods with varying biomechanical properties.

## Introduction

With an incidence of 12–15 cases per 10,000 inhabitants [1, 2] proximal ulna fractures account for 8–10% of skeletal injuries of the upper limb. In 80% of cases, these fractures are olecranon fractures that, in a broader context, belong to the category of osteoporotic fractures, and a further increase in their incidence can certainly be expected in the coming decades. All of these fractures are intra-articular in nature, so the ultimate goal is to restore joint function. Anatomic repositioning, retention, and early mobilisation are recommended as the current standard of care for virtually all proximal ulna fractures and yield the best results [3, 4]. Currently, the most widely used technique for open repositioning and internal fixation is the use of tension band wires (K-wires) and cerclage. However, a significant limitation of this technique arises in comminuted fractures [4–6]. Its principle relies on achieving interfragmentary compression, which is often unachievable with extensive comminution where stable fragment abutment necessary for this mechanism is lost, potentially leading to inadequate stabilization [3]. Consequently, plating techniques, which allow for multi-fragmentary fixation, are generally considered the standard approach for comminuted olecranon fractures with articular surface involvement, as supported by clinical experience and biomechanical evidence [7, 8]. On the other hand, osteosynthesis in this location requires the use of the smallest necessary osteosynthetic material because of the anatomical conditions and minimal amount of subcutaneous tissue. Furthermore, while locking plate technology is well-established, the optimal screw configuration within these plates for complex comminuted proximal ulna fractures, particularly concerning the balance between achieving adequate stability and minimizing implant bulk, remains an area of ongoing clinical discussion and relatively underexplored in the biomechanical literature [9–11]. One way to perform a sufficient fixation assessment is to analyse the strain-stress states.

The initial stability and modulation of mechanical forces at the fracture site can significantly influence the healing rate. These factors determine whether the fracture will heal through a direct or indirect pathway and ultimately via intramembranous or endochondral ossification [12]. Primary healing can be obtained by means of absolute stability and results in direct bone formation and osteonal bridging of the fracture gap. Bridging with a locking compression plate (LCP) is one of the methods of relative stability. It promotes indirect healing, which relies on some instability at the fracture site, allowing for interfragmentary motion (IFM). This controlled axial IFM, typically within a range of 0.2 to 1.0 mm for diaphyseal fractures, creates mechanical strain at the fracture site [13]. Such strain is perceived by mesenchymal stem cells, stimulating their differentiation towards chondrogenesis and initiating the formation of a cartilaginous soft callus, which is subsequently replaced by bone through endochondral ossification [14]. Studies have quantified this beneficial effect; Wolf et al. reported an enhancing effect of axial IFM of 0.4 mm [15], and a recent scoping review by Griffin et al. confirmed positive effects on callus formation with small-to-moderate axial IFM (mean 0.54 mm, range 0.2–0.9 mm) [16]. While excessive IFM can disrupt healing, appropriate levels of motion, particularly in the early phase of soft callus production, are crucial for robust secondary bone healing, though its clinical optimization remains challenging [14].

The aim of this study was to investigate and analyse, via in silico experimental modelling, the mechanical behaviour of a comminuted proximal ulnar fracture fixed with a screw-locking LCP. The analysis is aimed at assessing the mechanical interaction between the fragment surfaces on the basis of the loading of the fixed fracture with the LCP. Furthermore, this study aimed to compare strain intensity and interfragmentary motion (IFM) in fragments required for bone healing [17], as well as stress on the LCP, across four fixation variants. The analysis utilizes a computational modelling approach based on the now well-established and proven Finite Element Method (FEM), which is currently one of the most widely used methods for modelling the mechanical interaction between tissue and implants [18, 19]. This method also enables the creation of computational models of complex shapes and the solution of different variants and configurations within the system, which cannot be achieved through in vivo or in vitro experimentation today. To the best of the authors’ knowledge, few studies addressed clinical problem of this type using computational models of this scope.

## Materials and methods

The computational model of the elbow joint with fracture was created on the basis of typical fracture planes [20] (fracture type 2U1C3 by AO classification of proximal ulna fracture), selected with respect to clinical practice, to be as close as possible to real anatomical and pathological conditions. The position of the elbow joint was determined on the basis of the typical neutral position of the affected area after the surgical procedure [21]. The screw choices and number of screws were selected on the basis of clinical experience with respect to the recommendation of the AO Foundation [22]. The analysis of stress and strain was performed via the finite element method in software ANSYS^®^ Academic Research Mechanical, Release 22.2 (Swanson Analysis, Inc., Houston, PA, USA).

### Model of geometry

**Fig. 1:**
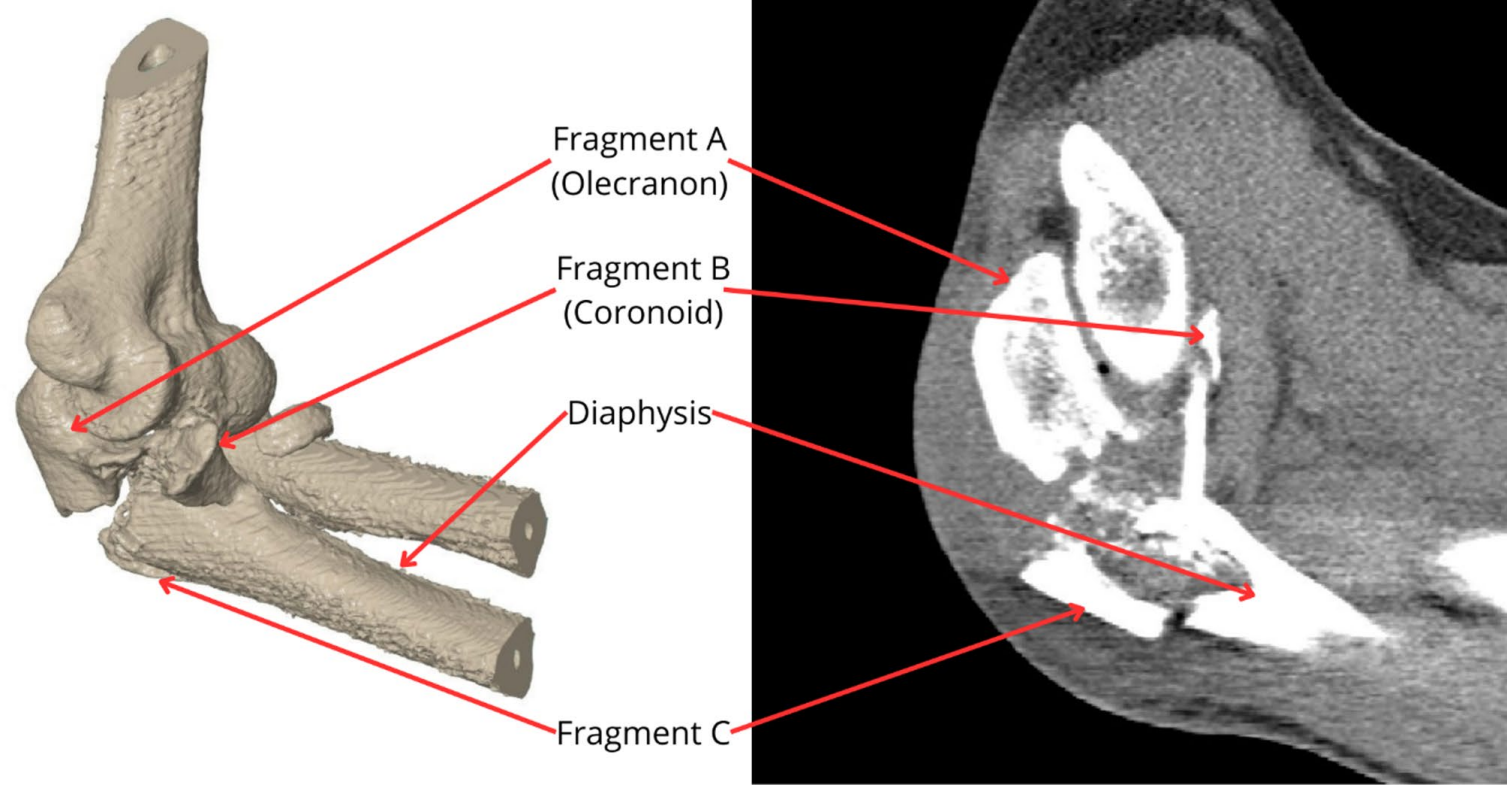
Analysing fracture of the ulna (left side– Model of the elbow joint with comminuted fracture; right side– CT image of the comminuted ulna)

For the purpose of this study two sets of images were used, which were obtained from Trauma Hospital Brno, Czech Republic (see Fig. 1). CT images of a fractured ulna (0.3 × 0.3 × 0.4 mm resolution) from a 50-year-old male patient were used to segment the geometry and spatial orientation of the characteristic fracture planes (type 2U1C3 by AO classification), enabling the reconstruction of individual comminuted fragments. This specific fracture morphology was then meticulously mapped onto a model of an intact, healthy ulna (derived from a separate CT dataset, 0.23 × 0.23 × 1 mm resolution). The CT datasets were converted into a standard tessellation language (STL) format file to acquire a geometry model of the elbow. All necessary image processing procedures were performed via MATLAB R2022b (Math Works, Natick, MA, USA) [23]. This approach combined a realistic fracture pattern with a standardized, healthy baseline bone geometry. Alignment and integration of the fracture planes onto the healthy ulna model were performed in Catia V5 (Dassault Systèmes, France) by registering key anatomical landmarks (e.g., olecranon tip, coronoid process, radial notch) common to both morphologies.

**Fig. 2:**
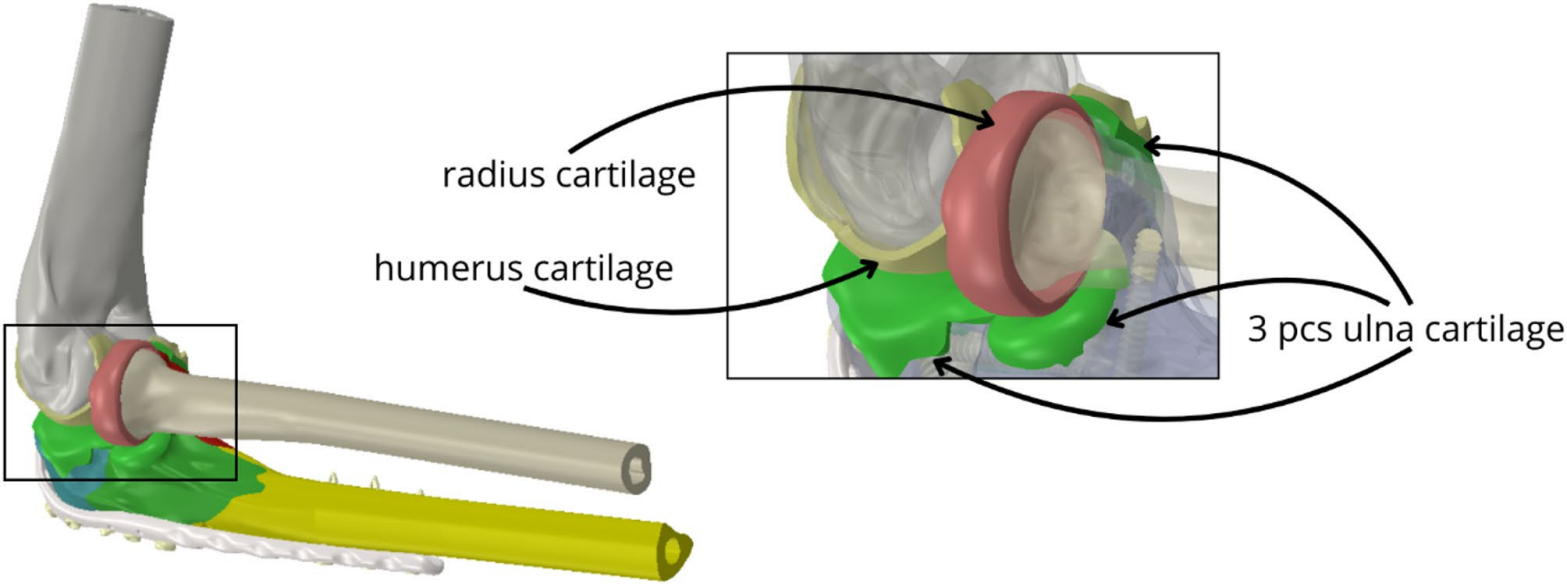
Complete model of geometry with detail of cartilages models

Cartilages were created by extending the articular surfaces into space by 1 mm. This value was chosen as a representative average based on consultation with an anatomist and literature data [24], acknowledging that cartilage thickness can vary regionally. For this comparative study, a uniform thickness was deemed a reasonable simplification (Fig. 2).

**Fig. 3:**
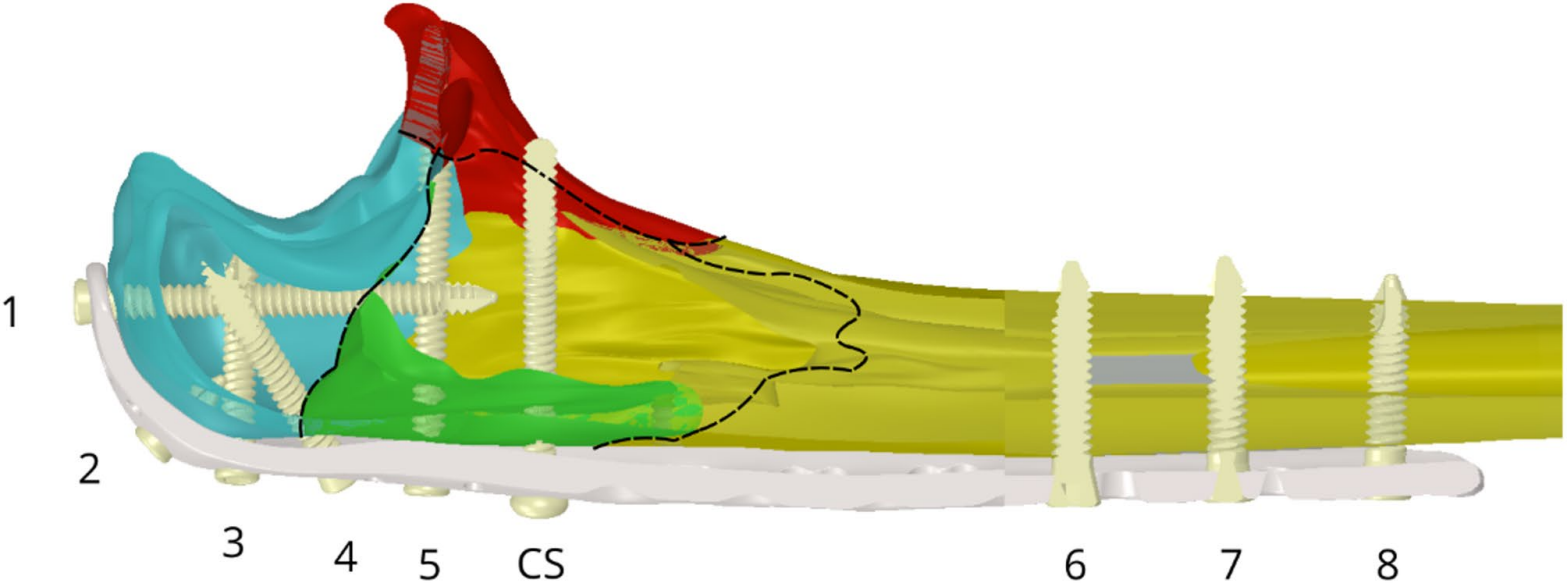
The ulna with the position of the screws and visualisation of the fragment edges

**Fig. 4:**
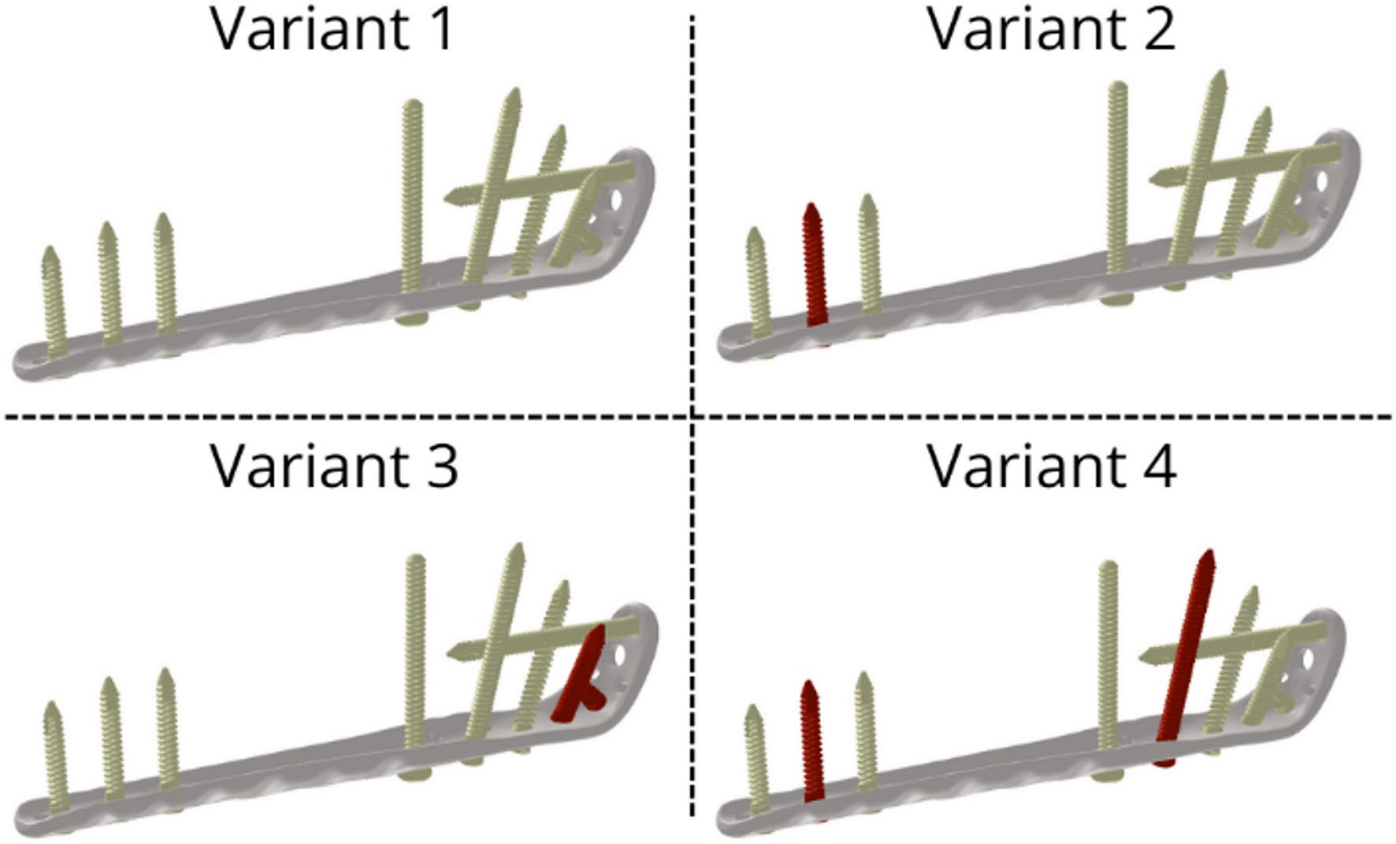
All variants of screws with plate (missing screws coloured red). Variant 1 includes all fixation screws. Variant 2 omits the middle bicortical screw from the distal fragment (screw 7, as per Table 1). Variant 3 omits the screws that anchor the proximal fragment of the olecranon (screws 2 and 3, as per Table 1). Variant 4 is configured like Variant 2 (omitting screw 7) but additionally omits the screw that holds the coronoid fragment in place (screw 5, as per Table 1)

Owing to the complexity of the LCP geometry, the LCP was scanned via a 3D scanner (Shining3D EinScan SE, SHINING 3D Technology GmbH, Stuttgart, Germany) and converted to a model geometry. The locking screws and cortical screw were measured and modelled in Catia V5 (Dassault Systèmes, France). The plate was attached to the fracture fragments via 9 screws (8 with a locking mechanism and 1 cortical screw) with different dimensions (see Table 1). The screws and plate were placed in position according to the fracture planes (see Fig. 3). For the purpose of assessing the analysed parameters in various clinical situations, 4 variants of fracture fixation were created, which differ in the number of screws used (see Fig. 4).

**Table 1:** Dimensions of using screws in the model and variant missing

Screws	Diameter [mm]	Length [mm]	Screw omitted in variant
Locking screw 1	3.45	38	Not missing
Locking screw 2	3.45	6	Variant 3
Locking screw 3	3.45	20	Variant 3
Locking screw 4	3.45	26	Not missing
Locking screw 5	3.45	38	Variant 4
Locking screw 6	3.45	20	Not missing
Locking screw 7	3.45	20	Variant 2, Variant 4
Locking screw 8	3.45	18	Not missing
Cortical screw (CS)	3.4	36	Not missing

### Material model

Linearly elastic, isotropic, and homogeneous material models were used in this study, base on the literature (see Table 2). The Young’s modulus of cortical and cancellous bone tissue was calculated based on the Hounsfield units obtained from CT images, using the relationships found in the literature [25–28]. According to the manufacturer (Zimmer^®^ Universal Locking System [29]), a plate and screws with locking heads were made from stainless steel alloy (22-13-5), and a cortical screw without a locking mechanism was made from stainless steel (316 L) from the manufacturer MEDIN, a.s. (Information from MEDIN Traumatology Catalogue [30]).

**Table 2:** Material properties

Material	Young’s Modulus [MPa]	Poisson‘s ratio [-]	References
Cortical bone	17 560	0.325	[25, 26]
Cancellous bone	2100	0.3	[25, 27, 28]
Cartilage	50	0.45	[31]
Stainless steel (316 L)	193,000	0.25	[32, 33]
Stainless steel (22-13-5)	200,000	0.28	[34, 35]

### Loading and constraints

**Fig. 5:**
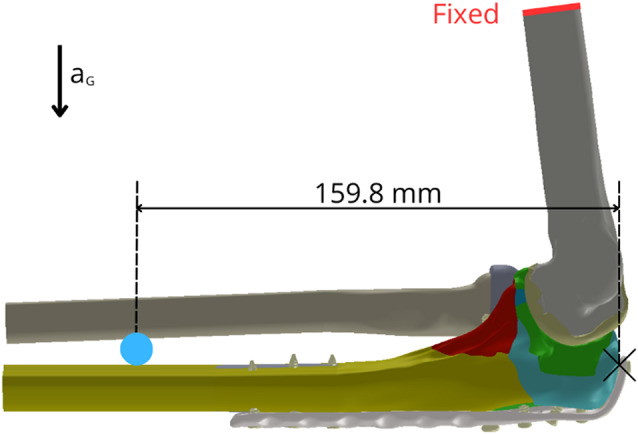
Visualisation of loads and constraints for the computational model (red line– fixed, blue point– mass 2.7 kg)

The FE model was subjected to loading conditions representing a stabilized, neutral post-operative position of the elbow joint. The primary external load was the self-weight of the forearm and hand, calculated as 2.7 kg based on anthropometric data (2.5% of total body weight for the forearm and 0.73% for the hand, assuming an average person of 83.6 kg [36, 37]). This mass, also accounting for the implant, was applied as a point mass located 159.8 mm distal to the olecranon tip (Fig. 5). Gravitational acceleration (9.81 m/s²) was applied to the entire model.

**Fig. 6:**
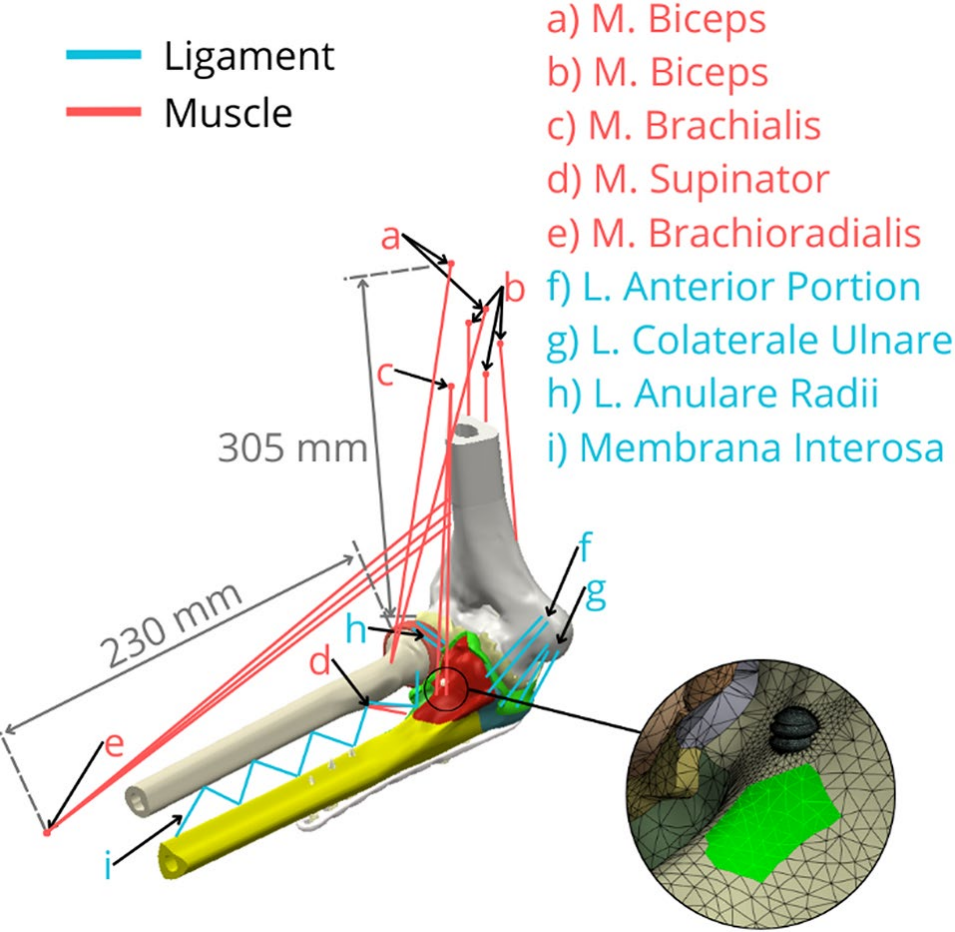
Visualisation positions of tendons for muscles and ligaments via the element LINK180 (blue lines– ligaments; red lines– muscles) with example of the tendon connection markings on the bone model

To simulate physiological joint stability and mimic a neutral post-operative state, constraints representing passive tension from major ligaments and isometric contraction of key muscle groups (e.g., triceps, brachialis, anconeus) crossing the elbow joint were incorporated [38]. These soft tissue structures were modelled using uniaxial tension-only spring elements (Ansys LINK180) and connected via remote points [39]. The locations and cross-sections of major muscle origins and ligament attachment points were determined based on standard anatomical texts [40], confirmed by consultation with an anatomist. These soft tissue constraints, through their pre-strain or defined stiffness, generate reaction forces acting on the forearm bones (ulna and radius), contributing to the overall stress state in conjunction with the applied self-weight [41]. The flat cut surface at the proximal end of the modelled humerus, as indicated in Fig. 5, was constrained in all translational degrees of freedom, providing fixed support for the entire model. The FE mesh for all components primarily consisted of ten-node tetrahedral elements (Ansys SOLID187) (Fig. 6).

**Fig. 7:**
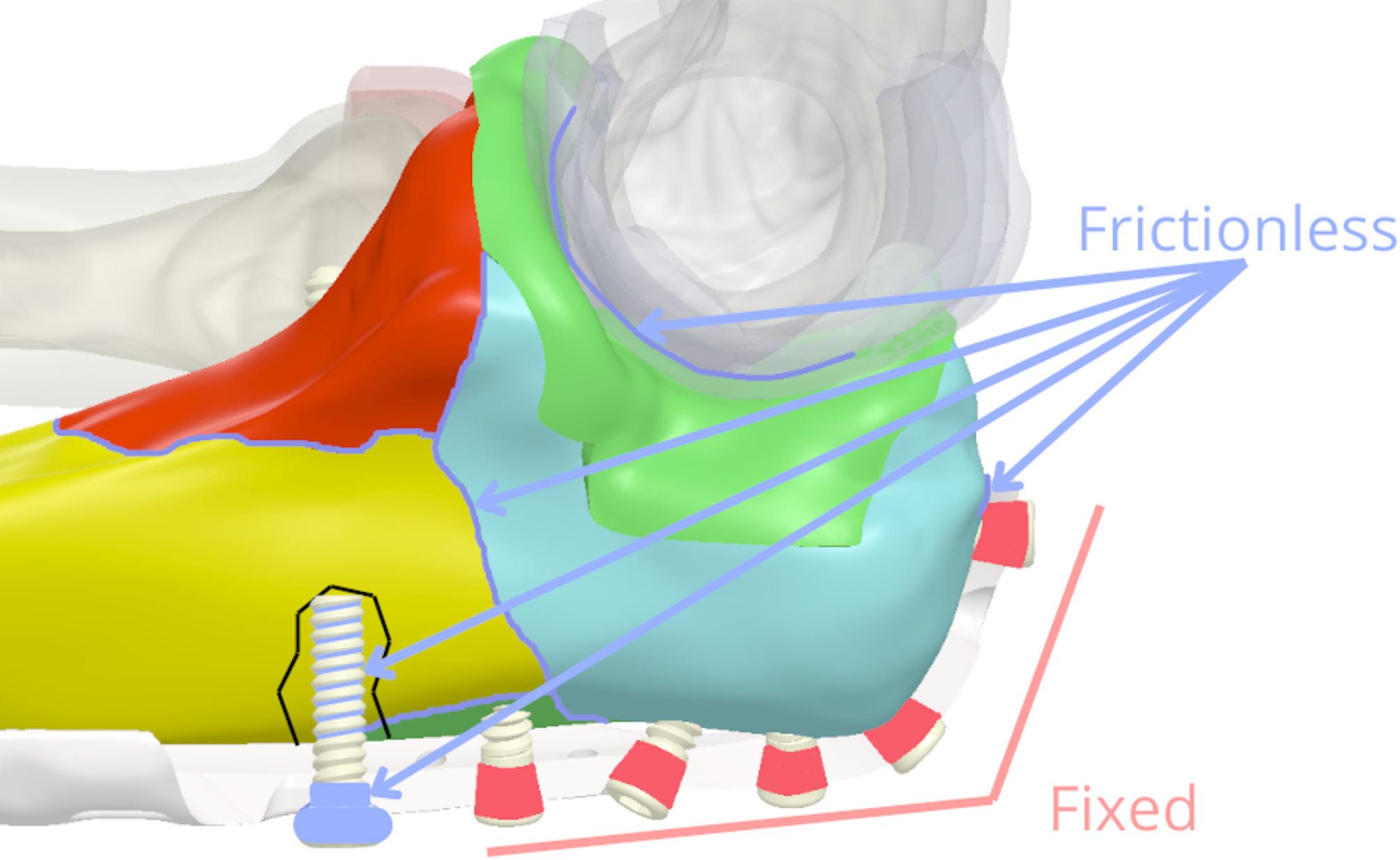
Proximal part of the ulna model with marked frictional and fixed areas

All parts in the model were connected using contact elements (Ansys elements TARGE170 and CONTA174), with a generally conservative approach taken for defining interactions to assess a scenario potentially maximizing stress on the implant and interfragmentary motion (IFM). Specifically, interfaces between distinct bone fragment surfaces, as well as between opposing articular cartilage surfaces, were modelled as frictionless to represent a worst-case scenario where frictional resistance does not contribute to stability. The interface between cartilage and its underlying cortical bone on the same anatomical part was modelled as bonded, reflecting their natural integration. For screw-bone interactions, the screw threads were defined as frictionless in relation to the surrounding bone tissue [42–44], representing an idealised immediate post-operative condition. Given the biological environment and the smoothness of the screws, we assume that the friction coefficient will probably be very low [45]. The cylindrical heads of all locking screws were also modelled as bonded to the plate. This set of contact definitions, particularly the ‘bonded’ assumption for locking screws in heads, represents an optimal screw anchorage scenario in both healthy and osteoporotic bone (Fig. 7).

**Fig. 8:**
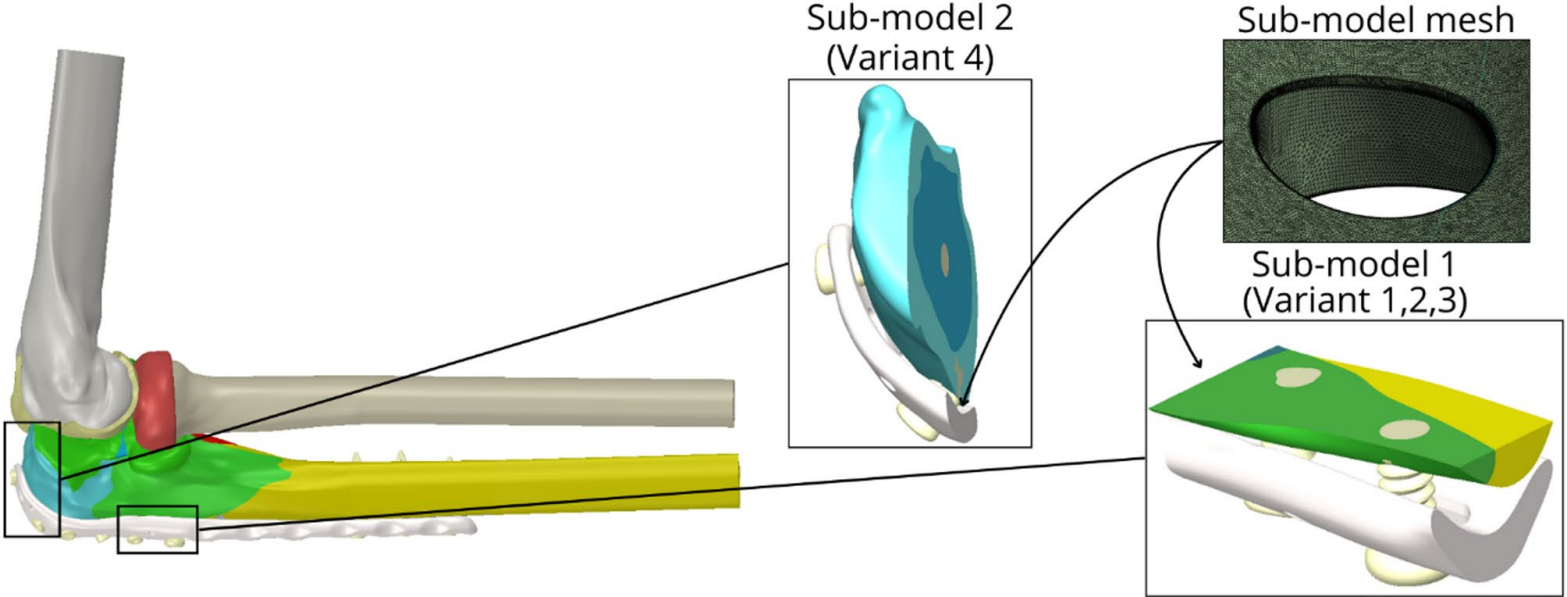
Model of geometry– used sub-models and visualisation of the sub-model mesh

The total number of elements ranged from approximately 1 418 000 to 1 500 000. Furthermore, a mesh sensitivity analysis was performed to determine the influence of the element size on the accuracy and convergence of the investigated parameters. For a more detailed analysis, a sub-modelling approach was employed. Sub-models were created in regions exhibiting the highest von Mises stress concentrations identified from the initial global analysis. These regions typically corresponded to areas around screw holes experiencing high load transfer or areas of the plate bridging significant gaps, which are clinically critical for implant integrity and potential failure (see Fig. 8). These sub-models had a total number of elements ranging from 1 130 000 to 1 350 000. (element sizes: rounding of holes– 0.02 mm, faces of holes– 0.1 mm, faces of plate around holes– 0.05 mm). The solved sub-models were analysed for parts of the LCP, bone tissue and screw geometry model for a specific region. Bone tissue was included in the sub-models because of the contact of bone tissue with the LCP.

**Fig. 9:**
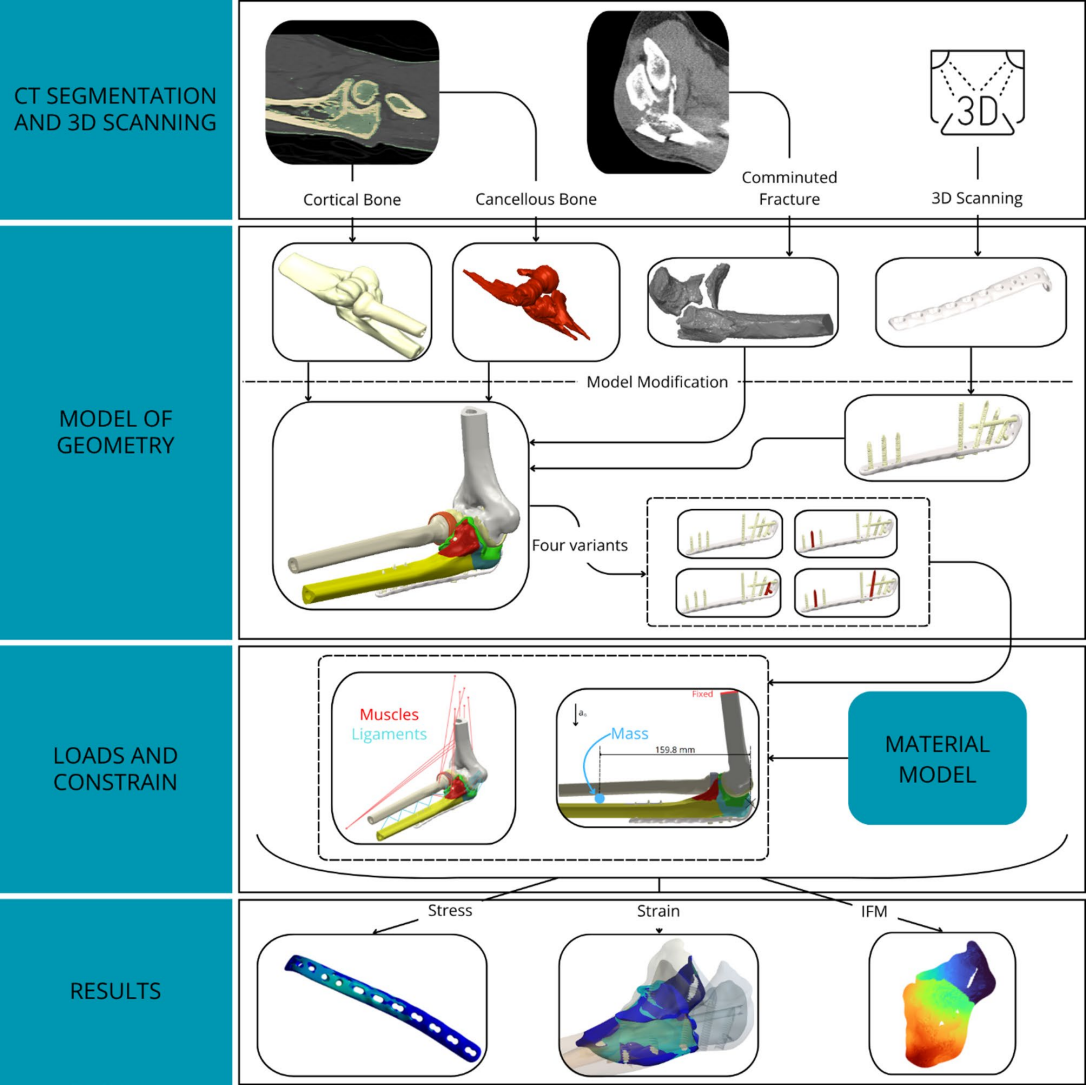
Workflow of the methodology

For improved clarity and transparency of the modelling procedure, an overview diagram of the study workflow is presented in Fig. 9. It summarises the key steps from data acquisition to result evaluation. This visual representation facilitates understanding of the methodology and enhances reproducibility.

### Evaluation

Using equivalent von Mises stresses, the critical spots on the plate were assessed, and the strain values on the surface of the individual fragments were identified. Strain intensity and Frost’s mechanostat hypothesis [46] were used to assess and analyse the mechanical interaction of bone fragments. On the basis of the Frost mechanostat hypothesis, the evaluation was performed on the basis of the strain intensity. The calculation of strain intensity is represented by the following equation:

$${\varepsilon _i} = MAX\>\left| {\left( {{\varepsilon _1} - {\varepsilon _2}} \right),\left( {{\varepsilon _2} - {\varepsilon _3}} \right),({\varepsilon _3} - {\varepsilon _1})} \right|,$$

where $\:{\varepsilon}_{i}$ is the strain intensity and where $\:{\varepsilon}_{1}$, $\:{\varepsilon}_{2}$ and $\:{\varepsilon}_{3}$ represent the principal strains. The strain intensity is expressed in the results in terms of$\:\:{\varepsilon}$, where $\:0.001{\varepsilon}$ = 0.1% [47]. According to Mechanostat, bone tissue responds to mechanical strain within distinct zones: Disuse/Atrhopy: 0 to $\:100\:{\upmu\:}{\varepsilon}$; Disuse/Remodelling: 100 to $\:1000\:{\upmu\:}{\varepsilon}$; Adaptive State/Remodelling:1000 to $\:1500\:{\upmu\:}{\varepsilon}$; Physiological Overload: 1500 to $\:3000\:{\upmu\:}{\varepsilon}$; Pathological Overload: 3000 to $\:25000\:{\upmu\:}{\varepsilon}$; Fracture >$\:25000\:{\upmu\:}{\varepsilon}$.

Finally, the IFM (interfragmentary motion) between the fragments was monitored, which according to previous studies [48], has a positive effect on the healing of bone tissue under certain circumstances. The IFM-Calculator software [49] in the Python development environment was used to determine the IFM.

## Results

**Fig. 10:**
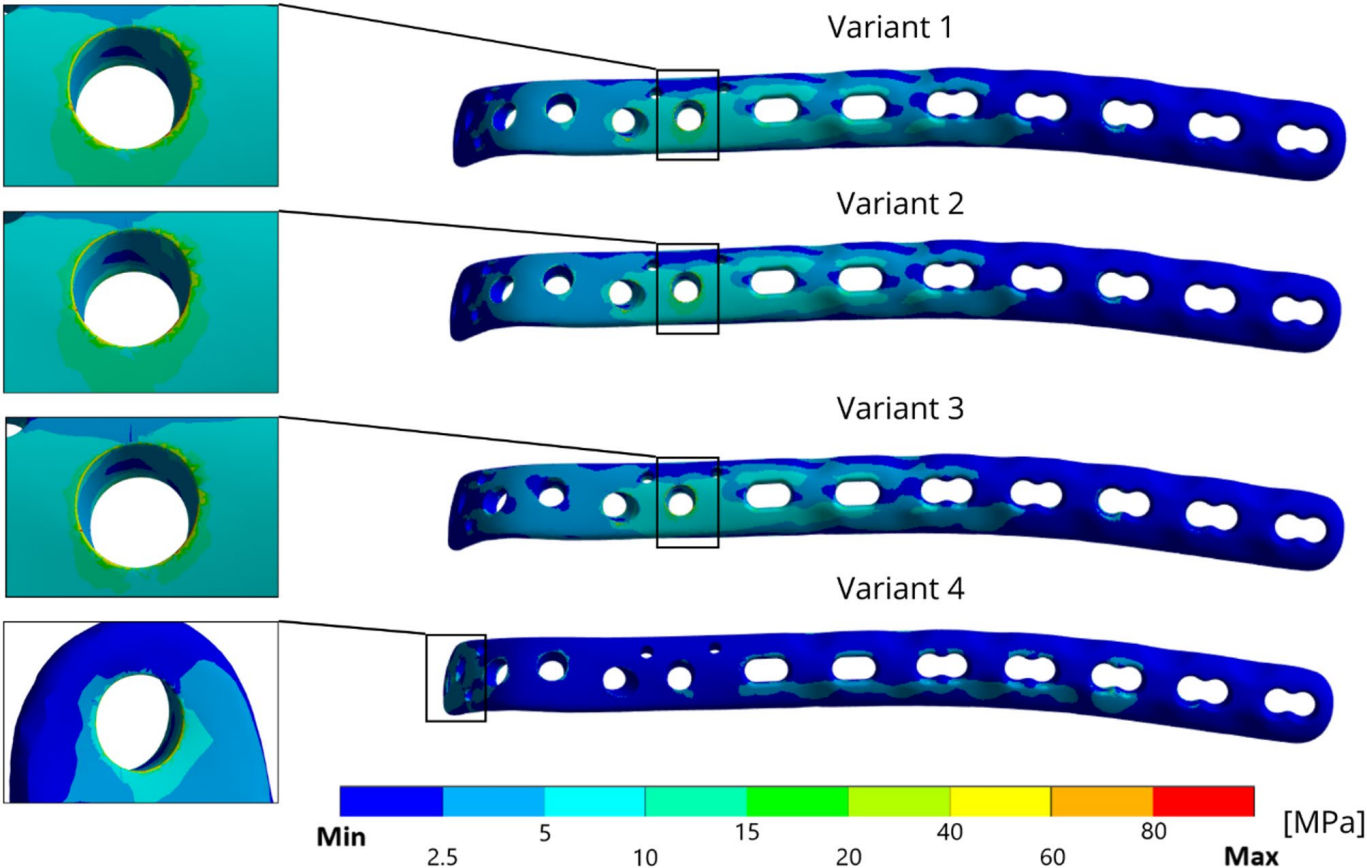
Distribution of von Mises stress for all the variants (focused areas have the highest equivalent stress distribution)

Figure 10 shows the distributions of the equivalent von Mises stresses on the plate for all the solved variants. For variants 1–3, the highest stress is in the area of locking screw 5 (see details in Fig. 10). The highest values of the equivalent stress range from 90 MPa to 120 MPa for variant 1. The lowest equivalent stress on the plate is in the case of variant 4, which lacks a locking screw (screw 5) and one screw in the diaphysis of the ulna (screw 7). The maximal equivalent stress is 98 MPa in the area of locking screw 1, which is in the olecranon of the ulna bone.

**Fig. 11:**
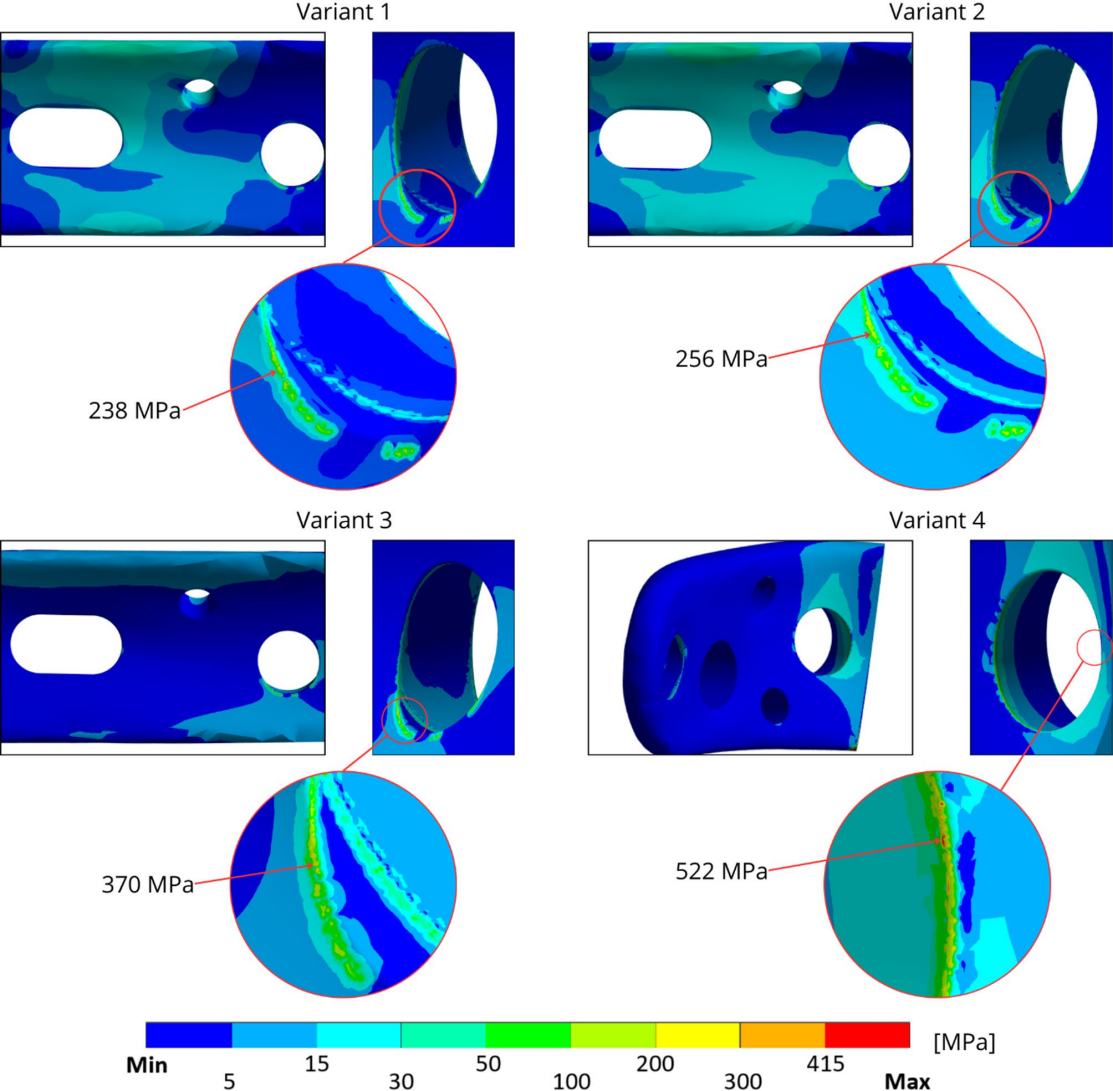
The von Mises stress distribution with sub-modelling results with its local maximum in each variant

The results from the sub-model for variants 1–3 in the locking screw 5 region are shown in Fig. 11, and the highest equivalent stress ranges from 200 MPa to 350 MPa for Variant 3. For Variant 4, the highest equivalent stress value is in the locking screw 2 region.

**Fig. 12:**
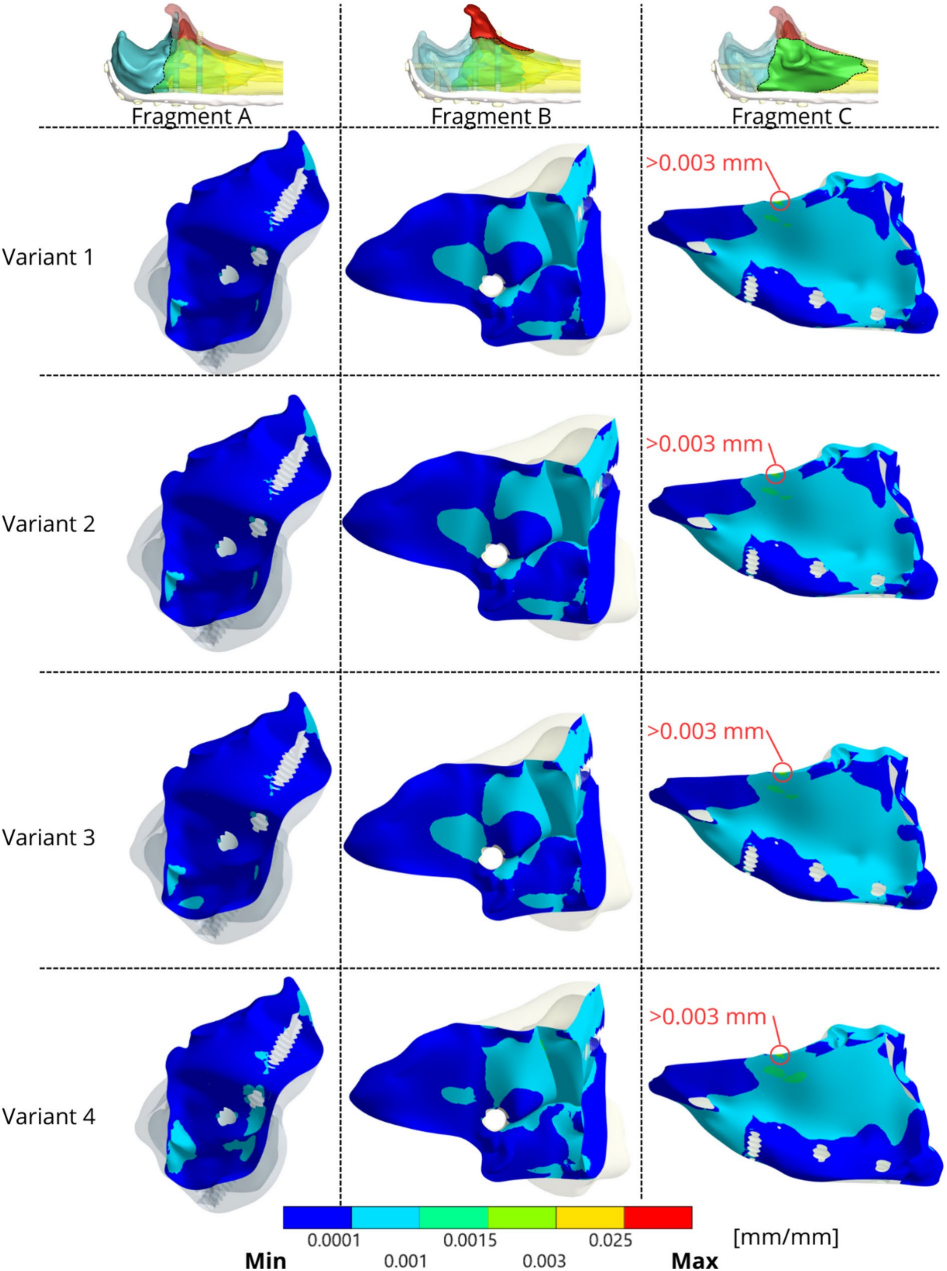
Strain intensity on each fragment on the fracture planes for each variant

Fig. 12 shows the equivalent strain intensity on the inner surface of the bone fragments. Fragment C near locking screw 5 is the fragment with the highest strain values, as it is the most mechanically loaded. The bone tissues near some screws exhibited strain intensities slightly exceeding $\:0.003\:{\varepsilon}$ ($\:3000\:{\upmu\:}{\varepsilon}$) on Fragment C, suggesting these areas are in the overload zone according to Frost’s Mechanostat hypothesis, potentially stimulating adaptive remodelling if sustained. The highest values of strain intensity in the bone tissues were observed in Variant 4.

**Fig. 13:**
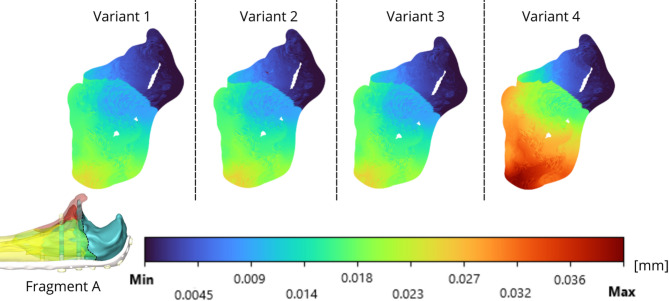
Olecranon fracture– IFM of fragment A

**Fig. 14:**
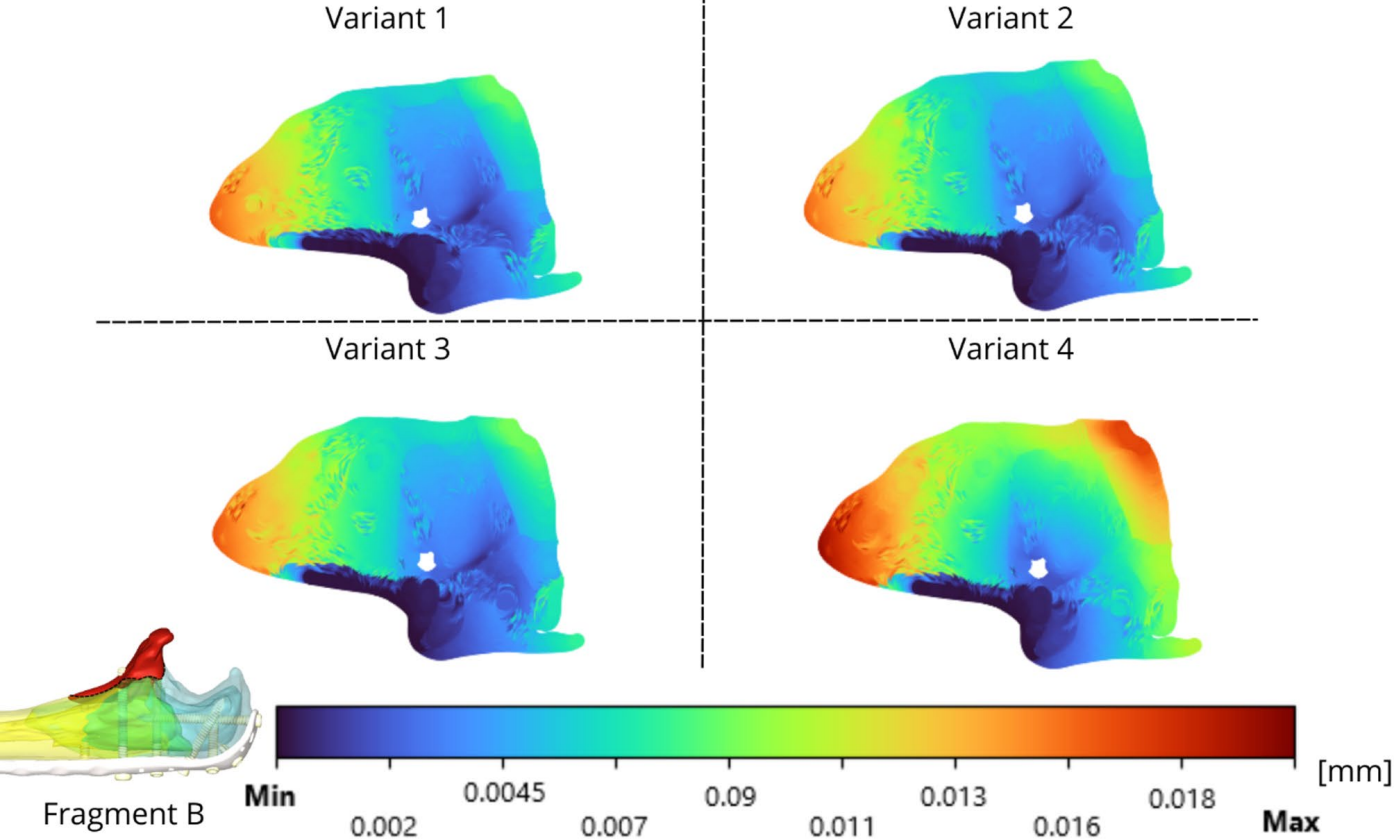
Coronoid fracture– IFM of fragment B

**Fig. 15:**
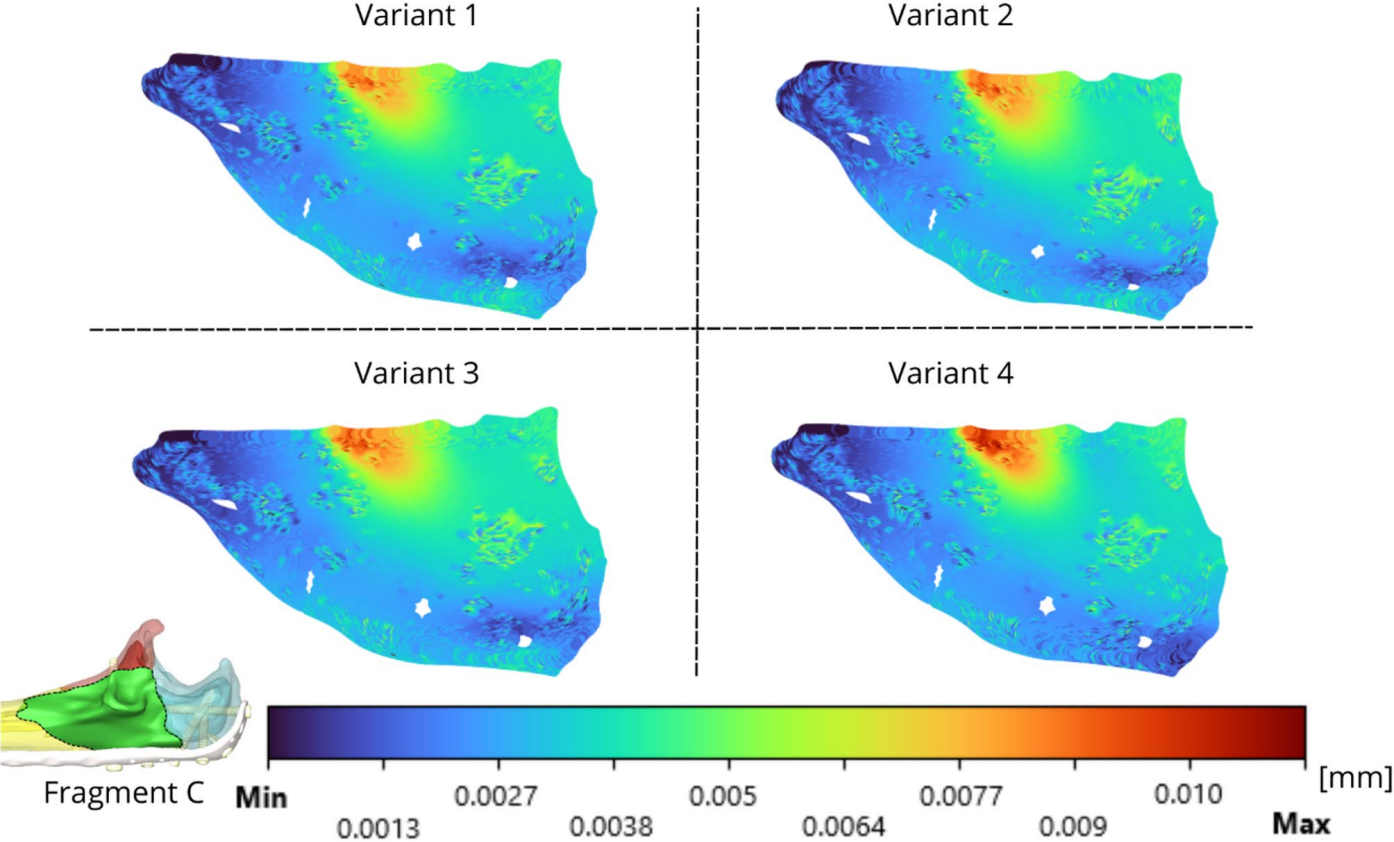
IFM of fragment C

The relative displacements are evaluated via IFM and are presented in Figs. 13-15 for the analysed fragments. The highest values of relative displacement are obtained in the case of Fragment A (olecranon), which is 0.041 mm. The highest displacements were always obtained in the case of Variant 4, as well as in the case of strain intensity analysed in bone tissue, and the other variants of screw insertion were comparable.

The Table 3 presents all evaluated mechanical outcomes, including stress in the plate, strain in the bone tissue, and interfragmentary motion for each fragment. To illustrate the effect of screw configuration changes, percentage differences are provided in comparison to Variant 1, which serves as the reference. These data enable a comprehensive assessment of the mechanical behaviour across all fixation strategies.

**Table 3:** Summary of mechanical outcomes and relative changes across fixation variants

Mech. Outcomes	Stress [MPa]		Strain [mm/mm]						IFM [mm]					
	LCP	%	Frag. A	%	Frag. B	%	Frag. C	%	Frag. A	%	Frag. B	%	Frag. C	%
Variant 1	157	0	0.00114	0	0.011	0	0.00092	0	0.02619	0	0.01698	0	0.01029	0
Variant 2	217	38.2	0.00114	0	0.012	9.1	0.00092	0	0.02704	3.2	0.01727	1.7	0.01033	0.4
Variant 3	370	135.6	0.00115	0.9	0.011	0	0.00096	4.4	0.02654	1.3	0.01752	3.2	0.01036	0.7
Variant 4	522	232.5	0.00149	30.7	0.012	9.1	0.00164	78.3	0.0408	56	0.02017	19	0.01153	12

## Discussion

Olecranon fractures are among the most common upper limb injuries. By definition, they are predominantly intra-articular fractures that have been treated surgically for decades, with the primary goal of achieving optimal functional outcomes. Surgical management typically adheres to the fundamental principles established by expert organisations, e.g. the AO Foundation [22]. This has led to the development and adoption of various alternative osteosynthesis techniques in addition to two conventionally established methods. Nevertheless, the literature reports a high incidence of complications and secondary surgeries in this context [5, 6, 50]. Some of these complications are associated with inadequate fracture healing and are often influenced by inappropriate fixation related to the number, direction and length of locking screws used [51].

In silico experiments using a computational model that incorporates all relevant anatomical and biomechanical parameters may help predict and prevent such complications. In our model, a complex fracture with characteristic fracture lines was intentionally selected [51]. This study examined how the number of screws affects LCP loading, bone remodelling, and interfragmentary motion (IFM), with the aim of evaluating and comparing bone healing outcomes within the context of a common clinical challenge: achieving stable fixation in comminuted proximal ulna fractures while potentially reducing implant material. The optimal configuration and number of screws, especially for controlling small fragments with pre-contoured plates [9] and achieving ideal construct stiffness, remain topics of clinical debate [10, 11]. Four screw configuration variants were developed, and comparative biomechanical analyses were performed.

The equivalent von Mises stress on the LCP was assessed across all the screw configurations. Due to the nature of loading, only static stress was analysed in the neutral postoperative position of the ulna, with high-cycle fatigue being excluded [20]. The analysis was conducted with respect to the standard healing period of 3–6 months [52]. The Sub-models were used to focus on the regions with the highest stress concentrations. For Variants 1–3, the maximum stress occurred near locking screw 5. The stress values ranged from 200 MPa to 350 MPa, with the highest observed in Variant 3, where screws 2 and 3—anchoring the olecranon—were omitted. In Variant 4, the stress concentration shifted toward the olecranon due to the absence of screw 5, where local plasticisation exceeding 415 MPa was observed in the screw hole region [53].

The LCP maintains the relative position of the bone fragments [52], allowing slight interfragmentary movement, which generates varying loads on the bone tissue. The equivalent strain intensity results indicate moderate loading of the bone tissue, which is consistent with physiological strain and normal bone remodelling due to fragment interactions. Most strain values ranged from 0.0001 to 0.0015 mm/mm, suggesting a healthy remodelling response. None of the configurations exceeded an equivalent strain of 0.025 mm/mm, which is indicative of pathological bone overloading or potential fracture strain [54, 55].

Fixation stability was also evaluated through analysis of the IFM. In all the tested configurations, the IFM values suggested sufficient stability for successful fracture healing [56, 57]. Variants 1–3 presented comparable IFM values, whereas Variant 4 presented the highest IFM, particularly in the olecranon region (Fragment A).

Although the IFM in our model reached a maximum value of only 0.041 mm, which is below the frequently cited threshold of 0.2–1.0 mm typically associated with optimal conditions for secondary bone healing through endochondral ossification [12, 58], this finding does not necessarily indicate impaired healing. Eghan-Acquah et al. [39] reported a similar IFM magnitude (approximately 0.04 mm) in their finite element model of a mandibular fracture treated with a flexible osteosynthesis plate and noted that such micromotion may still stimulate callus formation. This interpretation is consistent with the mechanoregulation theory by Prendergast et al. [59], which emphasizes the role of both tissue deformation and interstitial fluid flow in guiding tissue differentiation during fracture healing. In support of this, Zhang et al. [60] demonstrated that even relatively low interfragmentary displacement under partial weight-bearing conditions can create a mechanical environment favourable for endochondral bone formation. It is also important to consider that in periarticular regions such as the proximal ulna, primary bone healing with minimal callus formation is often the goal to preserve joint function. For primary healing, IFM values are generally expected to be significantly lower, often cited as below 0.03 mm [61]. While the maximum IFM of 0.041 mm observed in Variant 4 slightly exceeds this threshold, potentially posing a risk to ideal primary healing in that specific scenario, other configurations in our study generally maintained IFM levels more consistent with those conducive to primary bone healing or minimal, controlled callus formation.

Nonetheless, the IFM results highlight the importance of securely fixing the coronoid (Fragment B), potentially leading to fragment displacement under shear and compressive forces transmitted from the humerus, particularly during elbow flexion or forearm rotation, potentially compromising joint stability and congruity. Similarly, fixation of the apical fragment of the olecranon (Fragment A) is critical and typically ensured by standard plate designs.

It should be emphasized, however, that the interpretation of IFM results must be made with caution, as these numerical thresholds derived from literature have not been validated specifically for this fracture type and fixation construct through direct in vitro or clinical outcome data within our research. Given the challenges in conducting extensive experimental or clinical studies in such anatomically complex regions—where standardized mechanical testing is inherently difficult—finite element modelling represents a valuable and commonly accepted in silico approach for comparatively estimating biomechanical behaviour and guiding preclinical assessment of implant strategies.

Another limitation of this computational study is the use of a material model assuming linearly elastic, isotropic, and homogeneous properties for both cortical and cancellous bone tissues. While this simplification is frequently employed in comparative finite element analyses to assess mechanical interactions and relative performance of fixation constructs, it does not capture the inherent heterogeneity and anisotropy of bone. Although CT-based material property mapping can provide more patient-specific material distributions, it was not utilized here due to the necessary geometric repositioning of the bones from their scanned orientation to the neutral elbow position modelled in this study, which would invalidate direct voxel-to-element mapping. Furthermore, while advanced, CT-based mapping techniques also carry risks, such as the potential introduction of fictitious stress and strain concentrations if not implemented with extreme care, especially in regions of complex geometry or at material interfaces [62].

## Conclusion

This study evaluated four fixation variants for comminuted fractures of the proximal ulna, which differ in the number of screws used. The results of the stress and strain analysis revealed several important findings. The use of a 3.5 mm LCP and screws provides sufficient structural stability in this anatomical region. Given the recorded load levels, material failure is unlikely under the modelled loading conditions. Therefore, the use of less bulky implants may be both adequate and desirable, as it could reduce patient discomfort. Furthermore, while this study focused on the biomechanical performance of the fixation constructs, the subcutaneous nature of the olecranon makes implant prominence a critical clinical consideration. Surgeons must always aim to select implants and utilize techniques that minimize the hardware profile to reduce the risk of soft tissue irritation and patient discomfort, particularly under direct pressure or during elbow motion. However, the absence of fatigue analysis—for instance, considering repetitive flexion—represents a limitation of the present study and should be considered when interpreting the results.

The elevated bone loading around the screws further supports the established principle that screws should not engage directly with fracture lines. On the other hand, IFM analysis revealed that the presence of a coronoid fragment necessitates sufficient fixation with at least one screw to avoid exceeding the critical motion threshold, which could otherwise compromise articular healing or cause fragment detachment under early loading. This observation corresponds to clinical studies dealing with this typical injury [63–66].

The remaining IFM values observed across all the other configurations remained within the range favour able for primary bone healing, indicating that none of the studied constructs are excessively stiff.

It can be concluded that screw configurations 2 and 3, despite omitting some osteosynthetic material, still offered adequate fixation without significantly increasing the risk of failure in the studied case. Reducing the amount of material used can be advantageous due to cost savings, shorter operation times, and a design that is less cumbersome and better tolerated by patients.

The presented FE methodology, though applied to a specific comminuted fracture, is adaptable for patient-specific preoperative planning using individual CT data and can be extended to analyze other proximal ulna fracture patterns. Quantitative results, however, require cautious extrapolation due to inter-patient variability.

For future applications of this computational model, it would be valuable to compare various osteosynthesis techniques with differing biomechanical characteristics. Such comparative studies should be pursued in further research.

## Data Availability

Data is provided within the manuscript or supplementary information files. 10.5281/zenodo.15155804.

## References

[1] Court-Brown CM, Caesar B. Epidemiology of adult fractures: A review. Injury. Aug. 2006;37(8)691–697 10.1016/j.injury.2006.04.130 10.1016/j.injury.2006.04.130 16814787

[2] Duckworth AD, Clement ND, Aitken SA, Court-Brown CM, McQueen MM. The epidemiology of fractures of the proximal ulna. Injury. Mar. 2012;43(3):343–346. 10.1016/j.injury.2011.10.017 10.1016/j.injury.2011.10.017 22077988

[3] Wilkerson JA, Rosenwasser MP. Surgical techniques of olecranon fractures. J Hand Surg Am. Aug. 2014;39(8):1606–14. 10.1016/j.jhsa.2014.05.014. 10.1016/j.jhsa.2014.05.014 25070030

[4] Tarallo L, Mugnai R, Adani R, Capra F, Zambianchi F, Catani F. Simple and comminuted displaced olecranon fractures: A clinical comparison between tension band wiring and plate fixation techniques, Arch Orthop Trauma Sur. Aug. 2014;134(8):1107–1114. 10.1007/s00402-014-2021-9 10.1007/s00402-014-2021-9 24935660

[5] Qi H, et al. Comparison of clinical outcomes of three internal fixation techniques in the treatment of olecranon fracture: A retrospective clinical study. BMC Musculoskelet Disord. Dec. 2022;23(1):521. 10.1186/s12891-022-05482-8. 10.1186/s12891-022-05482-8 PMC9158155 35650582

[6] Powell AJ, Farhan-Alanie OM, McGraw IWW. Tension band wiring versus locking plate fixation for simple, two-part Mayo 2A olecranon fractures: A comparison of post-operative outcomes, complications, reoperations and economics, Musculoskelet Surg. Aug. 2019;103(2):155–160. 10.1007/s12306-018-0556-6 10.1007/s12306-018-0556-6 30006804

[7] Cutter B, Kelly SR, Beckett CR, Huish EG. The Biomechanical strength of olecranon fixation constructs: A systematic review and meta-regression. Int J Res Orthop. Feb. 2022;8(2):240. 10.18203/issn.2455-4510.IntJResOrthop20220614.

[8] Bethell MA et al. Tension band wiring and plate fixation for olecranon fractures– A systematic review and meta-analysis, JSES reviews, reports, and techniques, Jan. 2025, 10.1016/j.xrrt.2024.12.016 10.1016/j.xrrt.2024.12.016 PMC12277715 40697304

[9] Wellman DS, et al. Comminuted olecranon fractures: Biomechanical testing of locked versus minifragment non-locked plate fixation. Arch Orthop Trauma Surg. Sep. 2017;137(9):1173–9. 10.1007/s00402-017-2735-6. 10.1007/s00402-017-2735-6 28634743

[10] Anderson ML, Larson AN, Merten SM, Steinmann SP. Congruent elbow plate fixation of olecranon fractures. J Orthop Trauma. Jul. 2007;21(6):386–393. 10.1097/BOT.0b013e3180ce831e 10.1097/BOT.0b013e3180ce831e 17620997

[11] Wainberg SH, Moens NMM. The effect of working length, fracture, and screw configuration on plate strain in a 3.5 2-mm LCP bone model of comminuted fractures, VCOT Open, Jul. 2024;7(2):e117-e118 10.1055/s-0044-1787646

[12] Augat P, Simon U, Liedert A, Claes L. Mechanics and mechano-biology of fracture healing in normal and osteoporotic bone. Osteoporos Int. Mar. 2005;16:S36–43. 10.1007/s00198-004-1728-9. 10.1007/s00198-004-1728-9 15372141

[13] Klein P et al., The initial phase of fracture healing is specifically sensitive to mechanical conditions. Journal of Orthopaedic Research. Jul. 2003;21(4):662–669. 10.1016/S0736-0266(02)00259-0 10.1016/S0736-0266(02)00259-0 12798066

[14] Augat P, Hollensteiner M, von Rüden C. The role of mechanical stimulation in the enhancement of bone healing. Injury. Jun. 2021;52:S78–S83. 10.1016/j.injury.2020.10.009 10.1016/j.injury.2020.10.009 33041020

[15] Wolf S et al., The effects of external mechanical stimulation on the healing of diaphyseal osteotomies fixed by flexible external fixation. Clinical Biomechanics. Jun. 1998;13(4):–5, 359–364. 10.1016/S0268-0033(98)00097-7 10.1016/s0268-0033(98)00097-7 11415808

[16] Rechter GR, Anthony RT, Rennard J, Kellam JF, Warner SJ. The impact of early axial interfragmentary motion on the fracture healing environment: A scoping review., Injury. Dec. 2024;(55)12:111917. 10.1016/j.injury.2024.111917 10.1016/j.injury.2024.111917 39423671

[17] Miramini S, Zhang L, Richardson M, Mendis P, Oloyede A, Ebeling P. The relationship between interfragmentary movement and cell differentiation in early fracture healing under locking plate fixation. Australas Phys Eng Sci Med. Mar. 2016;39(1):123–33. 10.1007/s13246-015-0407-9. 10.1007/s13246-015-0407-9 26634603

[18] Du B, et al. Efficacy comparison of Kirschner-wire tension band and anchor loop plate in treatment of olecranon fracture. Front Bioeng Biotechnol. Sep. 2023;11. 10.3389/fbioe.2023.1203244. 10.3389/fbioe.2023.1203244 PMC10505394 37724095

[19] Yin N, Pan M, Li C, Du L, Ding L. The effect of ding’s screw and tension band wiring for treatment of olecranon fractures: a finite element study. BMC Musculoskelet Disord. Jul. 2023;24(1):603. 10.1186/s12891-023-06684-4. 10.1186/s12891-023-06684-4 PMC10364372 37488540

[20] Lubberts B, Mellema JJ, Janssen SJ, Ring D. Fracture line distribution of olecranon fractures. Arch Orthop Trauma Surg. Jan. 2017;137(1):37–42. 10.1007/s00402-016-2593-7. 10.1007/s00402-016-2593-7 27832347

[21] Morrey BF. Functional evaluation of the elbow. Morrey’s the elbow and its disorders. Elsevier; 2018. pp. 66–74. 10.1016/B978-0-323-34169-1.00005-X.

[22] Hodgson S, ‘AO Principles of Fracture Management’., The Annals of The Royal College of Surgeons of England. Jul. 2009;91(5):448–449. 10.1308/rcsann.2009.91.5.448b

[23] Marcián P et al., On the level of computational models in biomechanics depending on gained data from CT/MRI and micro-CT. Mendel. Jan. 2011:455–462.

[24] Rafehi S, Lalone E, Johnson M, King GJW, Athwal GS. An anatomic study of coronoid cartilage thickness with special reference to fractures. J Shoulder Elbow Surg. Jul. 2012;21(7):961–968. 10.1016/j.jse.2011.05.015 10.1016/j.jse.2011.05.015 21885303

[25] Rho JY, Hobatho MC, Ashman RB. Relations of mechanical properties to density and CT numbers in human bone. Med Eng Phys. 1995;17(5):347–55. 10.1016/1350-4533(95)97314-F. 7670694 10.1016/1350-4533(95)97314-f

[26] Cowin SC, Sadegh AM. Non-interacting modes for stress, strain and energy in anisotropic hard tissue. J Biomech. 1991;24(9):859–67. 10.1016/0021-9290(91)90311-A. 1752870 10.1016/0021-9290(91)90311-a

[27] Rho J-Y, Tsui TY, Pharr GM. Elastic properties of human cortical and trabecular lamellar bone measured by nanoindentation. Biomaterials. 1997;18(20):1325–1330. 10.1016/S0142-9612(97)00073-2 10.1016/s0142-9612(97)00073-2 9363331

[28] Reilly DT, Burstein AH, Frankel VH. The elastic modulus for bone. J Biomech. 1974;7(3):271–5. 10.1016/0021-9290(74)90018-9. 4846264 10.1016/0021-9290(74)90018-9

[29] Zimmer, Biomet. Zimmer^®^ Small Fragment Universal Locking System– Surgical Technique. Zimmer Biomet. Accessed: Jun. 02, 2025. [Online]. Available: https://www.zimmerbiomet.com/content/dam/zb-corporate/en/education-resources/surgical-techniques/specialties/trauma/zimmer-universal-locking-system/zimmersmallfragmentuniversallockingsystemsurgicaltechnique1.pdf

[30] Medin as, Instruments and, implants for traumatology. Medin, a.s. Accessed: Jun. 02, 2025. [Online]. Available: https://www.medin.cz/media/cache/file/08/TRAUMATOLOGIE-_-KATALOG-2024-_-14.10.2024.pdf.

[31] Peña E, Calvo B, Martínez MA, Doblaré M. A three-dimensional finite element analysis of the combined behavior of ligaments and menisci in the healthy human knee joint. J Biomech. 2006;39(9):1686–701. 10.1016/j.jbiomech.2005.04.030. 15993414 10.1016/j.jbiomech.2005.04.030

[32] Bevan MA et al. Mechanical Properties and Behavior of Additive Manufactured Stainless Steel 316L. 2017;577–583. 10.1007/978-3-319-51382-9_63

[33] Kang L, Chen F, Bradford MA, Liu X. Experimental study of mechanical properties of laser additively manufactured 316L stainless steels. Structures. Aug. 2023;54:221–235. 10.1016/j.istruc.2023.05.053

[34] Kusy RP, Dilley GJ. Materials Science Elastic Modulus of a Triple-stranded Stainless Steel Arch Wire via Three- and Four-point Bending. J Dent Res. Oct. 1984;63(10):1232–1240. 10.1177/00220345840630101401 10.1177/00220345840630101401 6592207

[35] Odegard BC, West AJ. On the thermo-mechanical behavior and hydrogen compatibility of 22-13-5 stainles steel. Mater Sci Eng. Jun. 1975;19(2):261–9. 10.1016/0025-5416(75)90113-5.

[36] Aydın, Tözeren. Human Body Dynamics. 2000 10.1007/b97432

[37] Walpole SC, Prieto-Merino D, Edwards P, Cleland J, Stevens G, Roberts I. The weight of nations: an Estimation of adult human biomass. BMC Public Health. Dec. 2012;12(1):439. 10.1186/1471-2458-12-439. 10.1186/1471-2458-12-439 PMC3408371 22709383

[38] Reed J, Bowen JD. Chapter 33 - Principles of sports rehabilitation. In: Seidenberg PH, Beutler AI, editors. The sports medicine resource manual. Philadelphia: W.B. Saunders; 2008. pp. 431–6. 10.1016/B978-141603197-0.10033-3.

[39] Eghan-Acquah E, et al. Enhancing Biomechanical outcomes in proximal femoral osteotomy through optimised blade plate sizing: A neuromusculoskeletal-informed finite element analysis. Comput Methods Programs Biomed. Dec. 2024;257:108480. 10.1016/j.cmpb.2024.108480. 10.1016/j.cmpb.2024.108480 39489075

[40] Netter FH. Atlas of human anatomy: classic regional approach. Volume 8. Elsevier; 2022.

[41] Nalbone L, et al. Study of a constrained finite element elbow prosthesis: the influence of the implant placement. J Orthop Traumatol. Apr. 2023;24(1):15. 10.1186/s10195-023-00690-x. 10.1186/s10195-023-00690-x PMC10102267 37055638

[42] Viceconti M, Muccini R, Bernakiewicz M, Baleani M, Cristofolini L. Large-sliding contact elements accurately predict levels of bone–implant micromotion relevant to osseointegration. J Biomech, Dec. 2000;33(12):1611–1618. 10.1016/S0021-9290(00)00140-8 10.1016/s0021-9290(00)00140-8 11006385

[43] Linetskiy I, Demenko V, Linetska L, Yefremov O. Impact of annual bone loss and different bone quality on dental implant success– A finite element study. Comput Biol Med. Dec. 2017;91:318–325. 10.1016/j.compbiomed.2017.09.016 10.1016/j.compbiomed.2017.09.016 29112907

[44] Korabi R, Shemtov-Yona K, Dorogoy A, Rittel D. The Failure Envelope Concept Applied To The Bone-Dental Implant System. Sci Rep. May 2017;7(1):2051. 10.1038/s41598-017-02282-2 10.1038/s41598-017-02282-2 PMC5435678 28515495

[45] Van Rietbergen B, Huiskes R, Weinans H, Sumner DR, Turner TM, Galante JO. The mechanism of bone remodeling and resorption around press-fitted THA stems. J Biomech. Apr. 1993;26(4):–5, 369–382. 10.1016/0021-9290(93)90001-U 10.1016/0021-9290(93)90001-u 8478342

[46] Marcián P, Wolff J, Horáčková L, Kaiser J, Zikmund T, Borak L. Micro finite element analysis of dental implants under different loading conditions. Comput Biol Med. Mar. 2018;96. 10.1016/j.compbiomed.2018.03.012. 10.1016/j.compbiomed.2018.03.012 29587150

[47] Frost H. ‘A 2003 update of bone physiology and Wolff’s Law for clinicians’, Angle Orthod. Mar. 2004;74:3–15. 10.1043/0003-3219(2004)074%3C0003:AUOBPA%3E2.0.CO;2 10.1043/0003-3219(2004)074<0003:AUOBPA>2.0.CO;2 15038485

[48] Yamaji T, Ando K, Wolf S, Augat P, Claes L. The effect of micromovement on callus formation. J Orthop Sci. 2001;6:571–5. 10.1007/s007760100014. 11793181 10.1007/s007760100014

[49] Sun J, Wu L, Fang N, Liu L. IFM calculator: an algorithm for interfragmentary motion calculation in finite element analysis. Comput Methods Programs Biomed. 2024;244:107996. 10.1016/j.cmpb.2023.107996. 38176328 10.1016/j.cmpb.2023.107996

[50] Bethell MA, et al. Complications associated with surgical management of olecranon fractures. JBJS Rev. Mar. 2025;13(3). 10.2106/JBJS.RVW.24.00163. 10.2106/JBJS.RVW.24.00163 40130946

[51] Norris BL, Lang G, Russell TA, Rothberg DL, Ricci WM, Borrelli J, editors. Absolute Versus Relative Fracture Fixation: Impact on Fracture Healing. J Orthop Trauma. Mar. 2018;32(3):S12–S16. 10.1097/BOT.0000000000001124 10.1097/BOT.0000000000001124 29461396

[52] Siebenlist S, Torsiglieri T, Kraus T, Burghardt RD, Stöckle U, Lucke M. Comminuted fractures of the proximal ulna—Preliminary results with an anatomically preshaped locking compression plate (LCP) system. Injury. Dec. 2010;41(12):1306–1311. 10.1016/j.injury.2010.08.008 10.1016/j.injury.2010.08.008 20828689

[53] Sumanariu CA, Amza CG, Baciu F, Vasile MI, Nicoara AI. Comparative Analysis of Mechanical Properties: Conventional vs. Additive Manufacturing for Stainless Steel 316L. Materials, Sep. 2024;17(19):4808. 10.3390/ma17194808 10.3390/ma17194808 PMC11478200 39410378

[54] Tyrovola JB, Odont X. The Mechanostat Theory of Frost and the OPG/RANKL/RANK System. J Cell Biochem. Dec. 2015;116(12):2724–2729. 10.1002/jcb.25265 10.1002/jcb.25265 26096594

[55] Frost HM. ‘Wolff’s Law and bone’s structural adaptations to mechanical usage: an overview for clinicians’, Angle Orthod. Jun. 1994;64(3):pp. 175–188. 10.1043/0003-3219(1994)064%3C0175:WLABSA%3E2.0.CO;2 10.1043/0003-3219(1994)064<0175:WLABSA>2.0.CO;2 8060014

[56] Perren SM. ‘Evolution of the internal fixation of long bone fractures: The scientific basis of biological internal fixation: choosing a new balance between stability and biology’, J Bone Joint Surg. Nov. 2002;84(8):1093–1110. 10.1302/0301-620X.84B8.13752 10.1302/0301-620x.84b8.13752 12463652

[57] Claes L, Augat P, Suger G, Wilke H. Influence of size and stability of the osteotomy gap on the success of fracture healing. Journal of Orthopaedic Research, Jul. 1997;15(4):577–584. 10.1002/jor.1100150414 10.1002/jor.1100150414 9379268

[58] Claes LE, Heigele CA. Magnitudes of local stress and strain along bony surfaces predict the course and type of fracture healing. J Biomech. Mar. 1999;32(3):255–66. 10.1016/S0021-9290(98)00153-5. 10.1016/s0021-9290(98)00153-5 10093025

[59] Prendergast PJ, Huiskes R, Søballe K. Biophysical stimuli on cells during tissue differentiation at implant interfaces. J Biomech. Jun. 1997;30(6):539–48. 10.1016/S0021-9290(96)00140-6. 10.1016/s0021-9290(96)00140-6 9165386

[60] Zhang L, et al. Computational modelling of bone fracture healing under partial weight-bearing exercise. Med Eng Phys. Apr. 2017;42:65–72. 10.1016/j.medengphy.2017.01.025. 10.1016/j.medengphy.2017.01.025 28236603

[61] Carrera I, Gelber PE, Chary G, González-Ballester MA, Monllau JC, Noailly J. Fixation of a split fracture of the lateral tibial plateau with a locking screw plate instead of cannulated screws would allow early weight bearing: a computational exploration. Int Orthop. Oct. 2016;40(10):2163–9. 10.1007/s00264-015-3106-y. 10.1007/s00264-015-3106-y 26780714

[62] Marcián P, et al. On the limits of finite element models created from (micro)CT datasets and used in studies of bone-implant-related Biomechanical problems. J Mech Behav Biomed Mater. May 2021;117:104393. 10.1016/j.jmbbm.2021.104393. 10.1016/j.jmbbm.2021.104393 33647729

[63] Jupiter Dringjb, Zilberfarb J, Posterior dislocation of the elbow with, Apr. fractures of the radial head and coronoid. The Journal of Bone and Joint Surgery-American Volume, 2002;84(4):547–551. 10.2106/00004623-200204000-00006 10.2106/00004623-200204000-00006 11940613

[64] Atwan Y, Arguello AM, Barlow JD. ‘Transulnar basal coronoid fractures– Surgical tips and tricks’, JSES Reviews, Reports, and Techniques. Aug. 2024;4(3):632–638. 10.1016/j.xrrt.2024.05.003 10.1016/j.xrrt.2024.05.003 PMC11329002 39157247

[65] Closkey RF, Goode JR, Kirschenbaum D, Cody RP. The Role of the Coronoid Process in Elbow Stability. The Journal of Bone and Joint Surgery-American Volume, Dec. 2000;82(12):1749–1753. 10.2106/00004623-200012000-00009 10.2106/00004623-200012000-00009 11130649

[66] Cha SM, Shin HD. Fixation of the Various Coronal Plane Fracture Fragments, Including the Entire Coronoid Process, in Patients with Mayo Type IIB Olecranon Fractures - Four Methods for Fixation. Indian J Orthop. Apr. 2019;53(2):224–231. 10.4103/ortho.IJOrtho_42_17 10.4103/ortho.IJOrtho_42_17 PMC6415574 30967689

